# Supplementary material for: Investigating causal networks of dementia using causal discovery and natural language processing models
Source: NPJ Dement. 2025 May 9;1(1):4. doi: 10.1038/s44400-025-00006-2 (PMC12064436; doi:10.1038/s44400-025-00006-2)
Supplement: Supplementary file 1 — Supplementary Information [file 44400_2025_6_MOESM1_ESM.pdf]

Supplementary Content

SUPPLEMENTARY CONTENT 1

Table S1 Full variable list for the study 3

Table S2 Pooled edge frequency table from network analysis with chronological age for 50 imputed datasets 3

Table S3 Pooled edge frequency table from network analysis with phenotypic age for 50 imputed datasets 3

Table S4 Variable index/annotations for the network analysis 3

Table S5 Variables selected by information retrieval models 3

Table S6 Dementia-related phrases input for information retrieval models 3

Table S7 Similarity scores for 5505 traits from word2vec model 3

Table S8 Similarity scores for 5505 traits from doc2vec model 3

Table S9 Phenotypic Age variables and weights 3

Figure S1. Venn diagram of selected phrases from two information retrieval models. 4

Figure S2. Partial pooled causal network including age 6

Figure S3. Partial pooled causal network including phenotypic age 7

Figure S4. Flowchart of variable selection using information retrieval models 9

Figure S5. Workflow of word2vec 11

Figure S6. Workflow of doc2ve 11

Figure S7. Missing percentage cut-off determination plot. 12

Note S1. Example of UKB variables pre-processing before information retrieval models: 13

Note S2. Illustration and examples of variable selection using information retrieval models 13

Figure S8. Cut-off determination plot. 15

Figure S9. Scatter plot of mean and standard deviation of similarity scores for each phrase. 16

Figure S10. Cut-off determination plot. 17

Note S3. Examples of the data pre-processing for selected variables 18

Note S4. Examples of sample quality control and the study cohort: 20

Note S5. Illustrations of the causal discovery approaches used in the study 21

Note S6. Interpretation of edges output from FCI algorithm [10] 21

Note S7. Python code for the variable selections 22

Note S8. R code for imputation and network analysis 25

References 27

Table S1 Full variable list for the study

Table S2 Pooled edge frequency table from network analysis with chronological age for 50 imputed datasets

Table S3 Pooled edge frequency table from network analysis with phenotypic age for 50 imputed datasets

Table S4 Variable index/annotations for the network analysis

Table S5 Variables selected by information retrieval models

Table S6 Dementia-related phrases input for information retrieval models

Table S7 Similarity scores for 5505 traits from word2vec model

Table S8 Similarity scores for 5505 traits from doc2vec model

Table S9 Phenotypic Age variables and weights

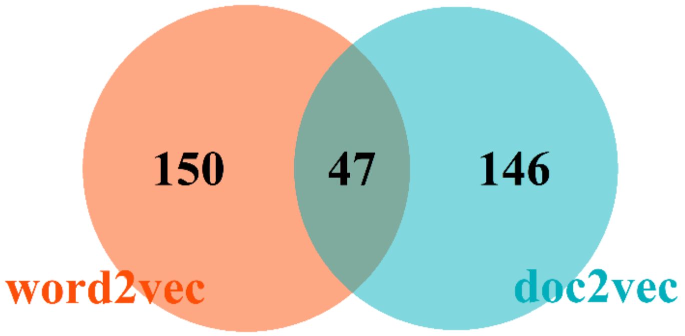

Figure S1. Venn diagram of selected phrases from two information retrieval models.

The origin circle represents phrases selected by word2vec model while the blue circle represents phrases selected by doc2vec models. Around ¼ of the phrases selected by two models are overlapped.

*Two traits with identical names identified by doc2vec was not covered by the figure.

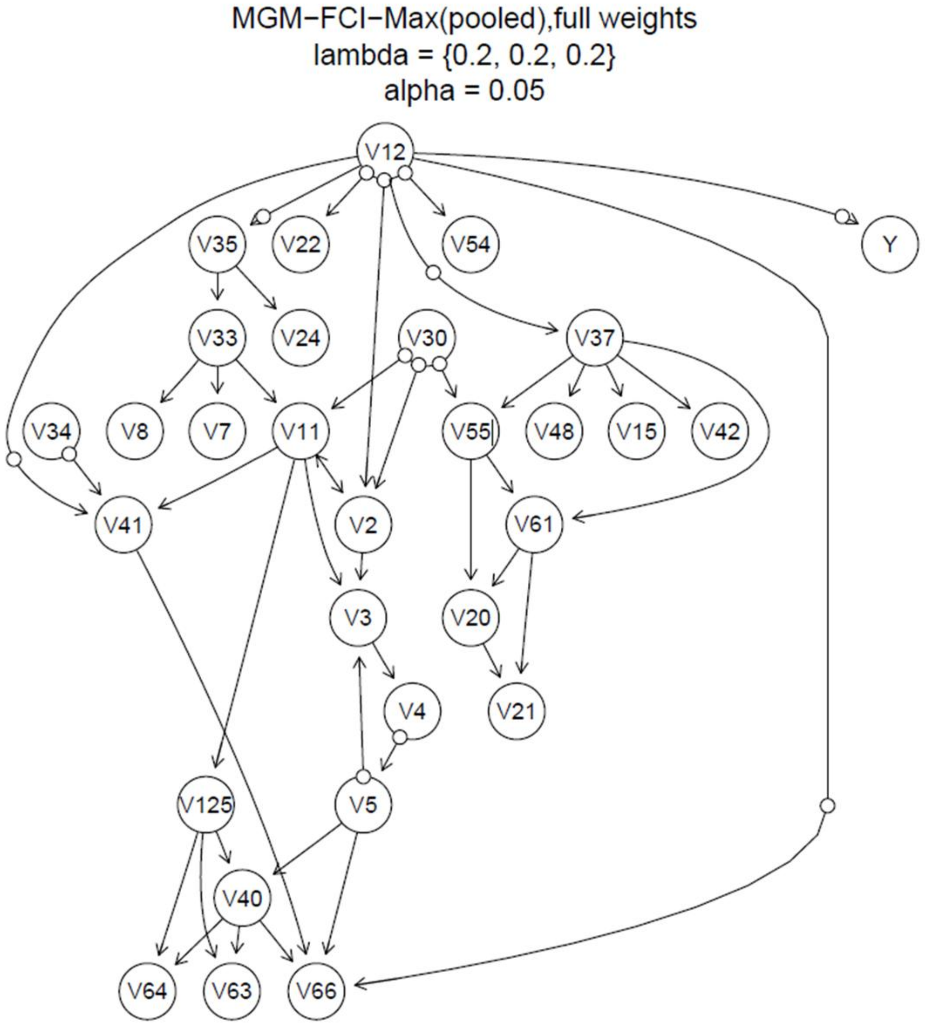

index	Phrase
V2	Basal metabolic rate
V3	HDL cholesterol_biochemistry
V4	Apolipoprotein A
V5	Cholesterol_blood
V7	Number of days/week of moderate physical activity 10+ minutes
V8	Number of days/week of vigorous physical activity 10+ minutes
V11	Body mass index (BMI)
V12	Age at recruitment
V15	Irritability
V20	Seen doctor (GP) for nerves, anxiety, tension or depression
V21	Seen a psychiatrist for nerves, anxiety, tension or depression
V22	Hearing difficulty/problems
V24	Prospective memory result
V30	Sex
V33	Physical_activity
V34	Medication for anti-inflammatories
V35	education
V37	Manifestations_of_mania_or_irritability
V40	diabetes_diagnosis
V41	vascular_heart_problems_diagnosis
V42	Cognitive symptoms severity over the past week
V48	Recent feelings or nervousness or anxiety
V54	Ever had bowel cancer screening
V55	Ever had prolonged feelings of sadness or depression
V61	Ever been offered/sought treatment for depression
V63	.E10.first.reported..insulin.dependent.diabetes.mellitus.
V64	.E11.first.reported..non.insulin.dependent.diabetes.mellitus.
V66	.E78.first.reported..disorders.of.lipoprotein.metabolism.and.other.lipidaemias.
V125	Glucose_pheno
Y	dementia_diagnosis

Figure S2. Partial pooled causal network including age

Presented causal associations in the graph are part of causal associations that are consistently inferred from all imputed datasets. In the graph, nodes represent variables (such as education), and edges (arrows) indicate causal links inferred from the Fast Causal Inference (FCI) algorithm. Directed edges suggest direct causal relationships between the variables. The presence of an "o" at the start of an edge signifies uncertainty, which could be due to potential unobserved confounding factors. For example, in a relationship denoted by A o– > B, it remains uncertain whether A directly causes B, if an unmeasured confounder influences both, or if a combination of these situations exists. Lambda value is the regularization parameters. Alpha value is the statistical threshold for significance, which is set to 0.05 in this study. The table on the right panel represents the annotations to the variables.

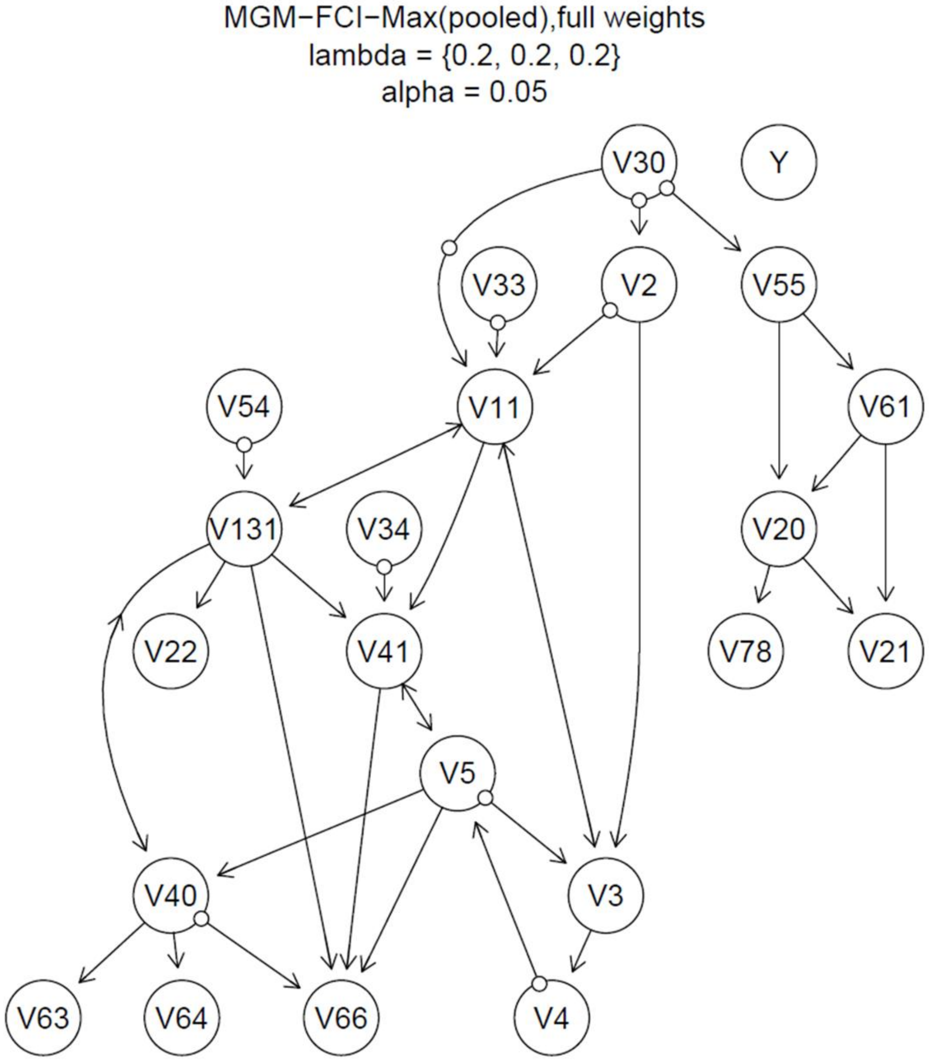

index	Phrase
V2	Basal metabolic rate
V3	HDL cholesterol_biochemistry
V4	Apolipoprotein A
V5	Cholesterol_blood
V11	Body mass index (BMI)
V20	Seen doctor (GP) for nerves, anxiety, tension or depression
V21	Seen a psychiatrist for nerves, anxiety, tension or depression
V22	Hearing difficulty/problems
V30	Sex
V33	Physical_activity
V34	Medication for anti-inflammatories
V40	diabetes_diagnosis
V41	vascular_heart_problems_diagnosis
V54	Ever had bowel cancer screening
V55	Ever had prolonged feelings of sadness or depression
V61	Ever been offered/sought treatment for depression
V63	.E10.first.reported..insulin.dependent.diabetes.mellitus.
V64	.E11.first.reported..non.insulin.dependent.diabetes.mellitus.
V66	.E78.first.reported..disorders.of.lipoprotein.metabolism.and.other.lipidaemias.
V78	.F41.first.reported..other.anxiety.disorders.
V131	phenoage
Y	dementia_diagnosis

Figure S3. Partial pooled causal network including phenotypic age

Presented causal associations in the graph are part of causal associations that are consistently inferred from all imputed datasets. In the graph, nodes represent variables (such as education), and edges (arrows) indicate causal links inferred from the Fast Causal Inference (FCI) algorithm. Directed edges suggest direct causal relationships between the variables. The presence of an "o" at the start of an edge signifies uncertainty, which could be due to potential unobserved confounding factors. For example, in a relationship denoted by A ⟜ > B, it remains uncertain whether A directly causes B, if an unmeasured confounder influences both, or if a combination of these situations exists. Lambda value is the regularization parameters. Alpha value is the statistical threshold for significance, which is set to 0.05 in this study. The table on the right panel represents the annotations to the variables.

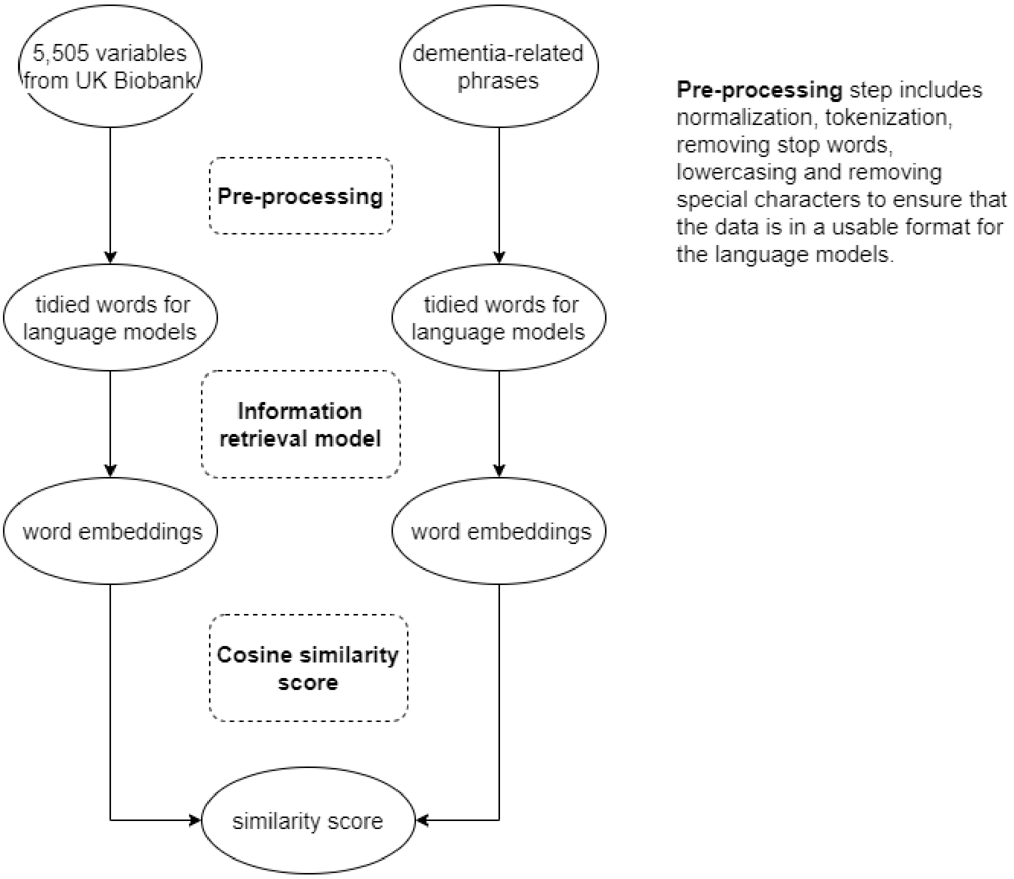

Figure S4. Flowchart of variable selection using information retrieval models

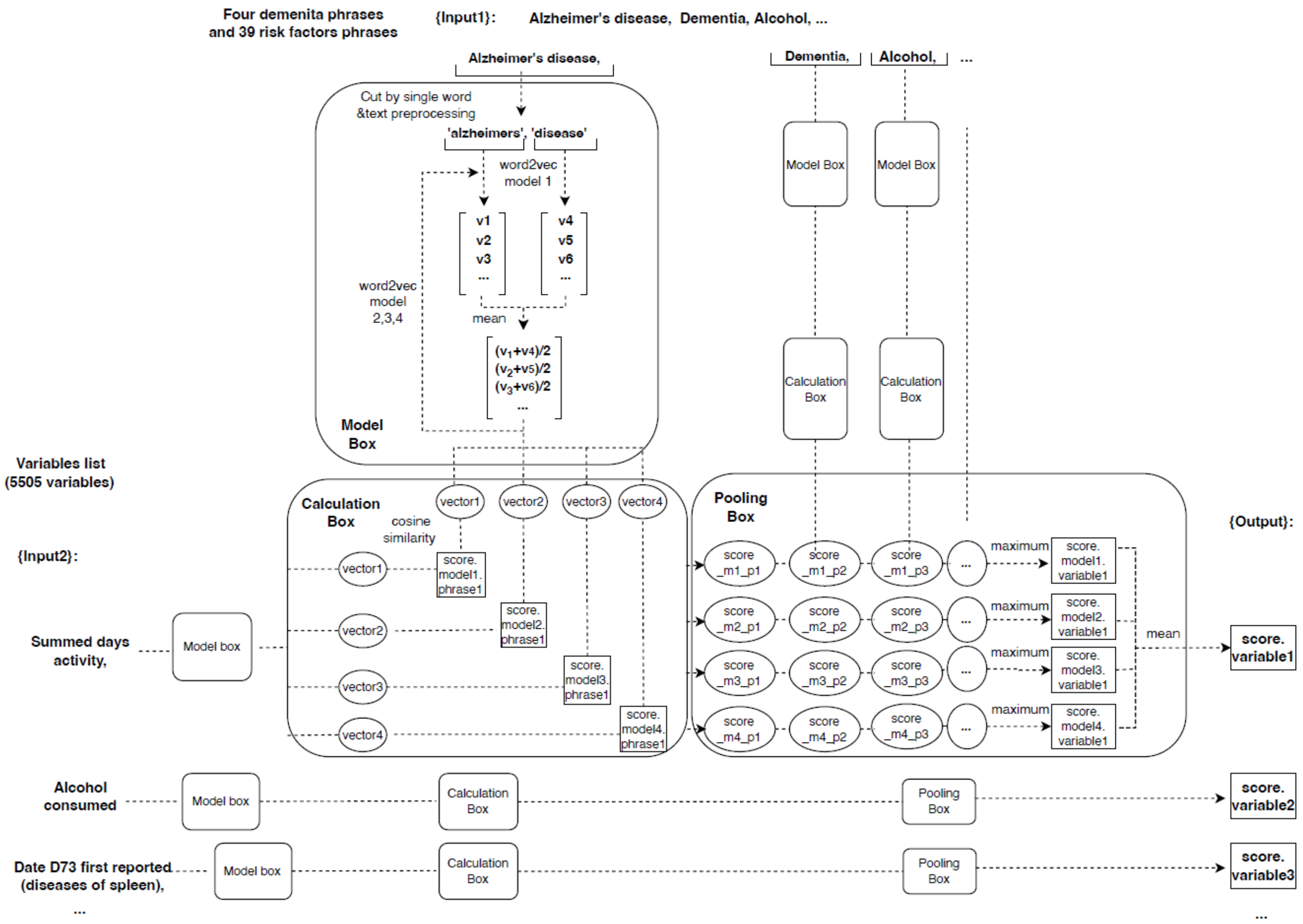

Four demenita phrases
and 39 risk factors phrases
{Input1}:
Alzheimer's disease, Dementia, Alcohol, ...
Alzheimer's disease,
Dementia,
Alcohol,
...
Cut by single word
&text preprocessing
'alzheimers', 'disease'
word2vec
model 1
v1
v2
v3
v4
v5
v6
word2vec
model
2,3,4
mean
(v1+v4)/2
(v2+v5)/2
(v3+v6)/2
Model
Box
Model Box
Model Box
Calculation
Box
Calculation
Box
Variables list
(5505 variables)
{Input2}:
Calculation
Box
cosine
similarity
vector1
vector2
vector3
vector4
score.
model1.
phrase1
score.
model2.
phrase1
score.
model3.
phrase1
score.
model4.
phrase1
Pooling
Box
score
_m1_p1
score
_m1_p2
score
_m1_p3
score
_m2_p1
score
_m2_p2
score
_m2_p3
score
_m3_p1
score
_m3_p2
score
_m3_p3
score
_m4_p1
score
_m4_p2
score
_m4_p3
maximum
score.
model1.
variable1
score.
model2.
variable1
score.
model3.
variable1
score.
model4.
variable1
mean
{Output}:
score.
variable1
Summed days
activity,
Model box
Alcohol
consumed
Model box
Calculation
Box
Pooling
Box
score.
variable2
Date D73 first reported
(diseases of spleen),
Model box
Calculation
Box
Pooling
Box
score.
variable3
...

Figure S5. Workflow of word2vec

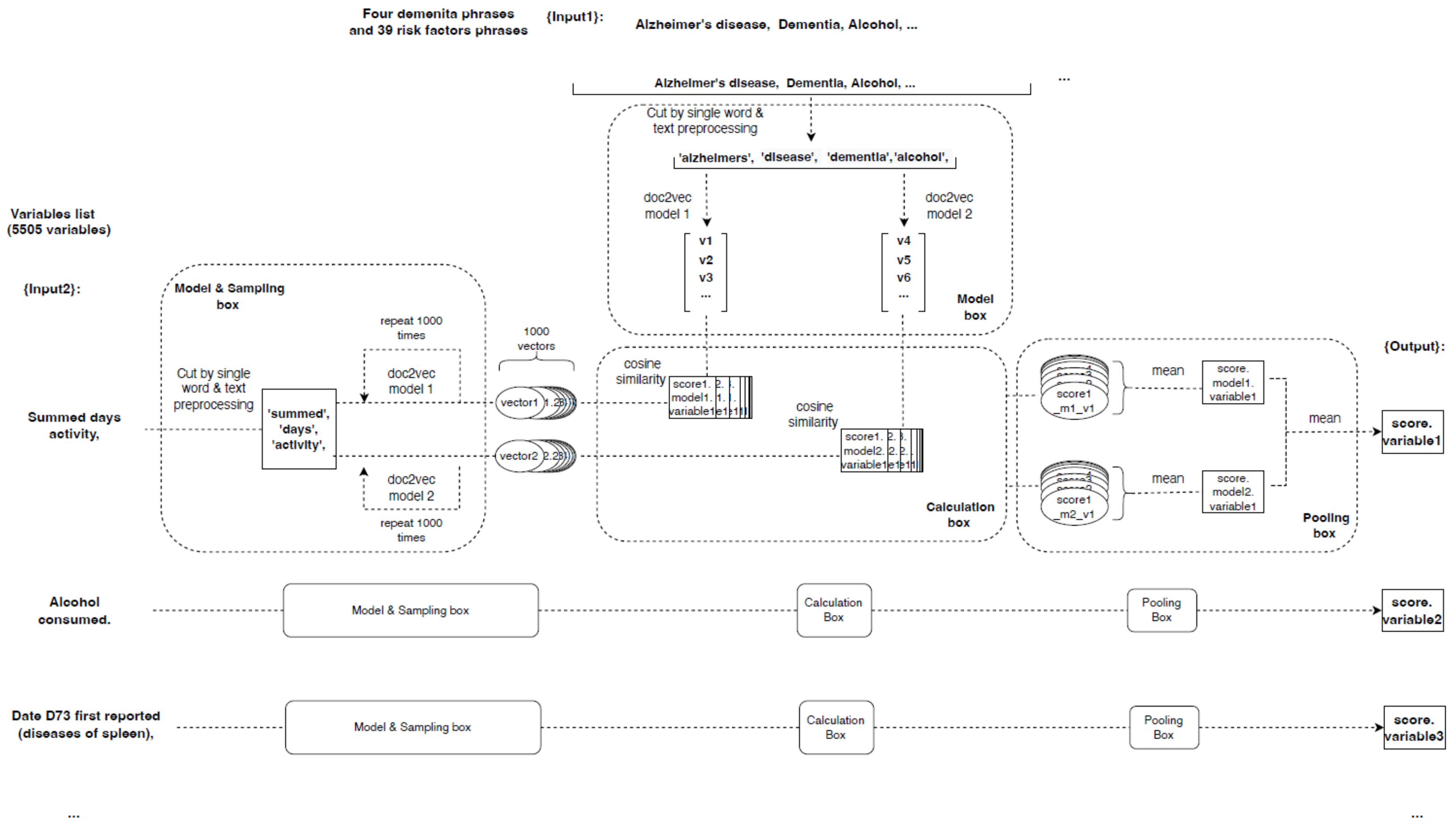

Figure S6. Workflow of doc2ve

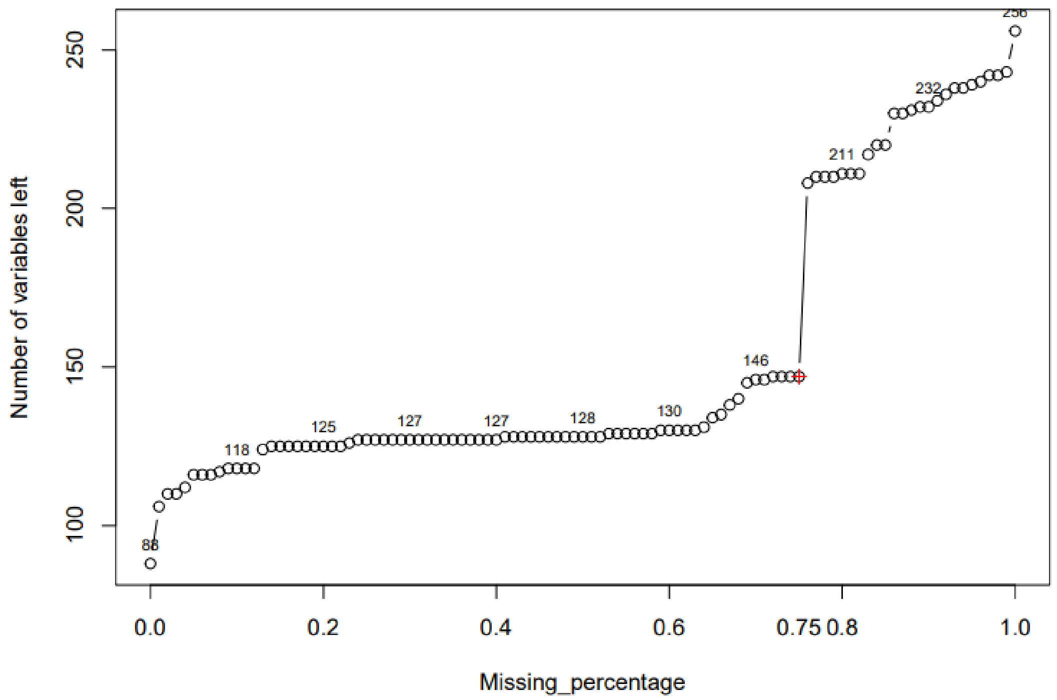

Figure S7. Missing percentage cut-off determination plot.
This plot shows the number of variables against different missing percentage thresholds. The X-axis is the threshold of missing rate, ranging from 0 to 1. The Y-axis is the number of variables that have missing rate below the threshold.

Note S1. Example of UKB variables pre-processing before information retrieval models:

We obtained the full variable list from UK Biobank data dictionary (n=9079), https://biobank.ctsu.ox.ac.uk/crystal/exinfo.cgi?src=accessing_data_guide. From the list, we filtered out irrelevant traits, the detailed steps are:

a. Filtered out:
    i. genomics (n=199)
    ii. imaging (n= 2687)
    iii. single-gender traits: single-gender stated in UK Biobank data dictionary (n= 45).
    iv. Health-related outcomes (n=2575): covid-19, primary care, hospital inpatient, death register, cancer register, algorithmically defined
    v. Health-related outcomes: dementia-related mental and behaviour disorders, F00-F03, G30 (https://biobank.ctsu.ox.ac.uk/crystal/refer.cgi?id=593)
    vi. single-gender ICD10 diseases:
        O00-O9A Pregnancy, childbirth and the puerperium (148 traits)
        P00-P96 Certain conditions originating in the perinatal period (92 traits)
        N40-N53 Diseases of male genital organs (24 traits)
        N70-N77 Inflammatory diseases of female pelvic organs (16 traits)
        N80-N98 Noninflammatory disorders of female genital tract (38 traits)

From the above steps, we obtained a variable list containing 5505 traits (**Table S1**) as potential variable pool for network analysis, of which 1932 ICD-diagnosis and the date it diagnosed +3573 baseline characteristics. These variables are then filtered by information retrieval models by variable names.

Note S2. Illustration and examples of variable selection using information retrieval models

The information retrieval (IR) models, based on algorithms like Word2Vec [1] and Doc2Vec [2], are designed to understand and quantify the relationships between ‘terms’. Word2Vec is a model that learn word embeddings – numerical representations capturing various features of words – from extensive text datasets. This model effectively maps words into a high-dimensional vector space, where each word is represented by a vector. The position and direction of these vectors are learned from the context in which words appear in the training dataset, allowing the model to capture nuances in word usage and meanings. Doc2Vec, an extension of Word2Vec [2], operates on a similar principle but at the document level. Rather than just focusing on individual words, Doc2Vec converts entire documents into vector representations. This allows for capturing the broader context and meaning of phrases and sentences, which is particularly useful when analysing complex texts. After transforming words and documents into vectors, we used the cosine similarity score to determine the similarity between the potential variable names and the target dementia-related phrases. Cosine similarity measures the cosine of the angle between two vectors in a multidimensional space. A score of 1 indicates maximum similarity, meaning that the two vectors are identical while a score of -1 representing maximum dissimilarity. This method enables us to quantitatively assess how closely the variables in our dataset are related to key terms associated with dementia.

Example of the workflow are:

2.1 Definition of the key phrases associated with dementia.

We included both dementia and its known modifiable risk factors as the aimed ‘dementia-related’ phrases. We defined the aimed phrases as “dementia”, “Alzheimer’s disease”, “cognitive decline” and “memory loss”, as well as 39 risk factors from literature [3] that are, "Bilingualism","Alcohol","Cognitive engagement","Diet","Physical activity","Sleep","Smoking","Social engagement","Stress","Arthritis","Atrial

fibrillation","Anxiety","BMI","Cancer","Carotid atherosclerosis","Cholesterol","Depression","Diabetes","Hearing loss","Homocysteine","Hormones","Hyper/hypotension","Inflammatory markers","Metabolic syndrome","Motor function","Peripheral artery disease","Renal disease","Serum uric acid","Stroke","TBI","Antacids","Antihypertensives","Anti-inflammatories","Benzodiazepines","HRT","Insulin sensitizers","Statins","Pesticides".

2.2 implementation of the algorithms

- Algorithms for similarity score calculated with word2vec:
  For each test phrase:
  1. Preprocessing the Phrase (Tokenization):
     1.1 Split the phrase into its individual words.
     1.2 Convert words to lowercase.
     1.3 Remove non-alphanumeric characters and non-relevant words (stopwords) such as "and", "the".
  2. Similarity score analysis:
     For Word2Vec Model 1 (and later Models 2, 3, 4):
     2.1 Obtain mean embedding:
         2.1.1 Convert each test phrase's token into a vector form (word embedding) using the current model.
         2.1.2 take the mean vector that encapsulate their meaning as the mean embedding for the test phrase.
     2.2 For each dementia-related phrase:
         2.2.1 Tokenize the dementia phrase in the same way as in step 1.
         2.2.2 Obtain the mean embedding for the dementia phrase same way as in step 2.1.
     2.3 Compute the cosine similarity score between the test phrase and the current dementia-related phrase.
     2.4 Retain the highest similarity score across different dementia phrases, indicating the closest relation of the test phrase to any of the dementia phrases using the current model.
  3. Result Compilation:
     Calculate the mean of the similarity scores obtained from all four models. The results indicate the relevance between the test phrase and dementia-related phrases over four word2vec models.

- Algorithms for similarity score calculated with doc2vec:
  For each test phrase:
  1. Preprocessing the Phrase (Tokenization):
     1.1 Convert words to lowercase.
     1.2 Remove non-alphanumeric characters and non-relevant words (stopwords) such as "and", "the".
  2. Similarity score analysis:
     For Doc2Vec Model 1 (and later Model 2):
     2.1 Tokenize all dementia phrases in the same way as in step 1.
     2.2 Convert all dementia phrase's tokens into a vector form (word embedding) using the current model.
     2.3 Random processing for each phrase:
         2.3.1 Convert the phrase tokens into a vector form (word embedding) using the current model.
         2.3.2 Compute the cosine similarity score between the test phrase and the current dementia-related phrase.
         2.3.3 Repeat step 2.3.1 and 2.3.2 for 1000 times and take the mean of all similarity scores as the final output.
  3. Result Compilation:

Calculate the mean of the similarity scores obtained from two four models. The results indicate the relevance between the test phrase and dementia-related phrases over two doc2vec models.

2.3 Results

1. Similarity scores from word2vec algorithms:
   In determining the optimal threshold for our analysis, we plotted the number of filtered results against different similarity score thresholds to determine the cut-off threshold (Figure S8). A lower threshold results in a larger set of variables, increasing the comprehensiveness of our results but potentially diluting the accuracy with less relevant findings. Conversely, a higher threshold imposes stricter criteria, yielding a more focused set of variables that likely enhances the precision of our model at the risk of excluding relevant variables. Choosing between the inclusivity and precision, we set the threshold to be 0.7 and selected 197 dementia-related traits from the original variable pool.

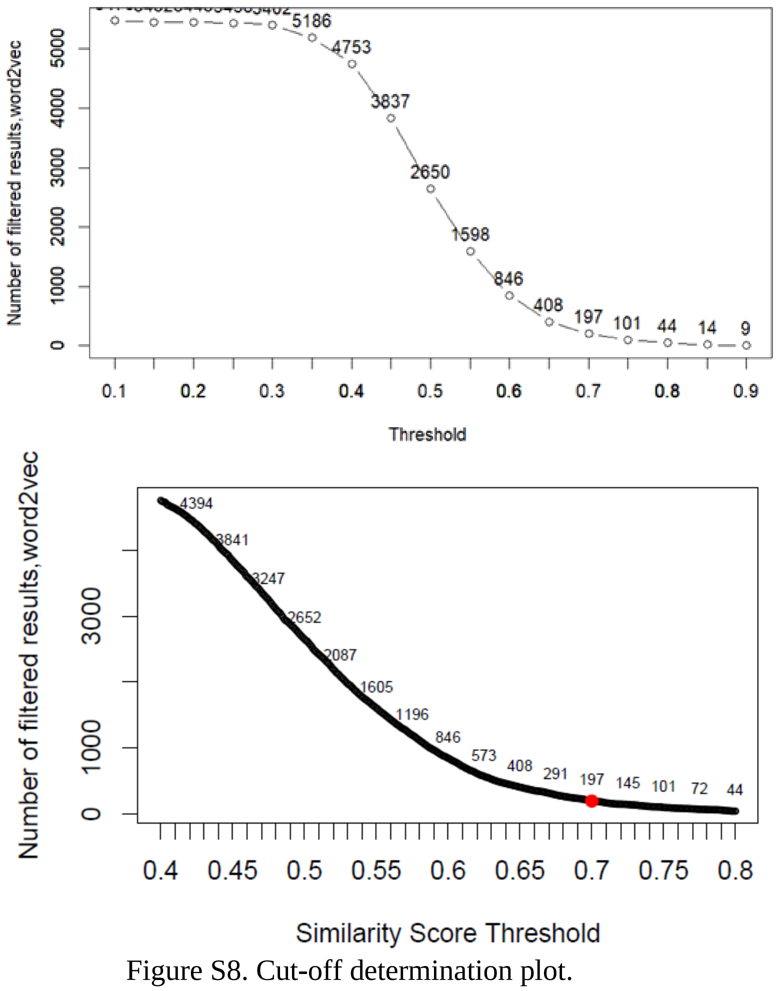

Figure S8. Cut-off determination plot.

This plot shows the number of filtered results against different similarity score thresholds. The X-axis is the threshold of similarity score, ranging from 0 to 1. The Y-axis is the number of traits that have similarity score above the threshold.

2. Similarity scores from Doc2Vec algorithms:
   The algorithms behind Doc2Vec models includes inherent randomness, and each vector is just the result of a progressive approximation process that settles on a 'good enough' vector [2]. This causes a slightly difference output in each time run. To make sure the random process has little effect on the output. We examined the mean and standard deviations values for each phrase’s similarity scores in 1000 runs. In the diagnostic plot (Figure S9), majority

of the results have a stable result with variance random distributed across mean values. Therefore, we took the mean similarity from the 1000 runs for each phrase.
The ouliers – Phrases with zero variance appeared to be not recorded in the model database, and therefore have zero similarity score and being ignored. We further filtered out the results with Signal-to-Noise Ratio (SNR) value < 10, which suggest the results are not stable.

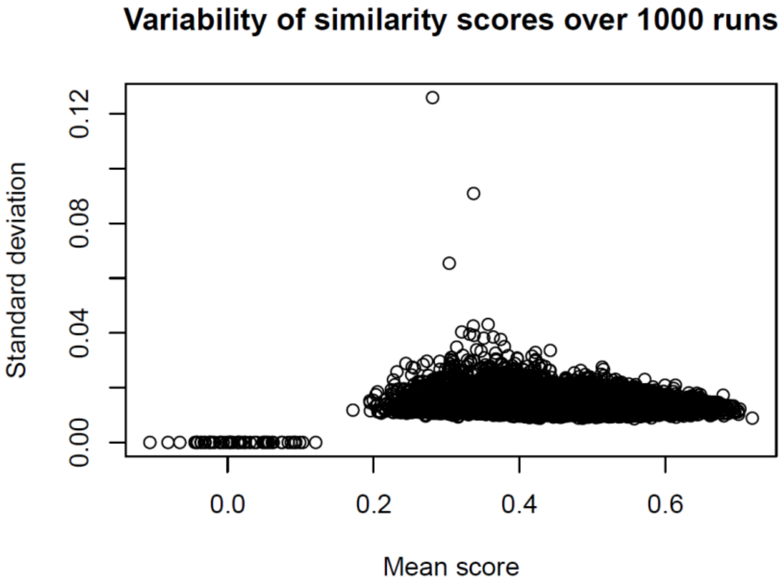

Figure S9. Scatter plot of mean and standard deviation of similarity scores for each phrase. The standard deviations are randomly located around mean, with no association or trend observed.

Again, we plotted the number of filtered results against different similarity score thresholds to determine the cut-off threshold (Figure S10). The threshold for similarity scores calculated by doc2vec models was set to be 0.58. 194 variables were selected by this threshold.

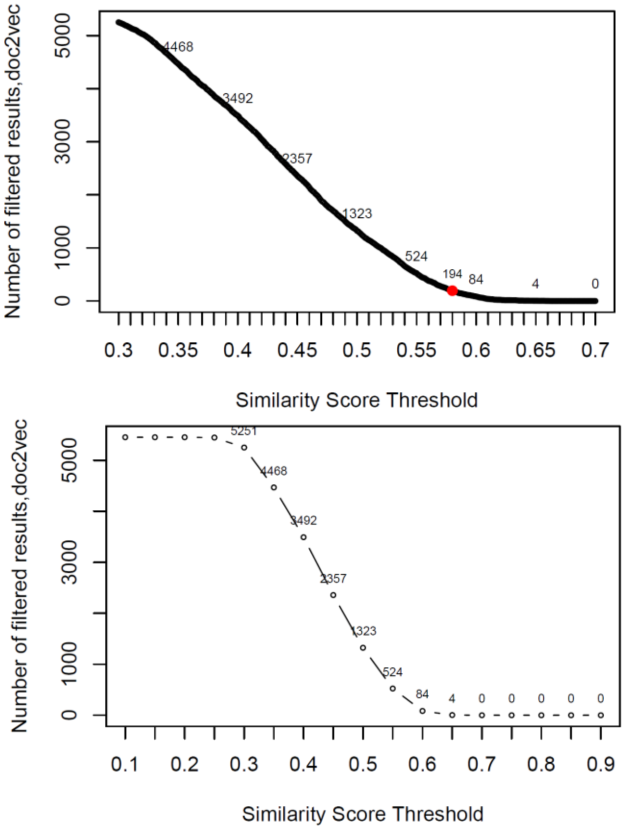

Figure S10. Cut-off determination plot.

This plot shows the number of filtered results against different similarity score thresholds. The X-axis is the threshold of similarity score, ranging from 0 to 1. The Y-axis is the number of traits that have similarity score above the threshold.

3. From similarity scores calculated by two IR models, we selected 344 traits were for downstream network analysis (Figure S1). The detail of the selected traits can be found in see supplementary **table S5.**

4. Ten variables that are not present in the variable pools:

"Bilingualism", "Homocysteine", "Hormones", "Serum uric acid", "Antacids", "Antihypertensives", "Benzodiazepines", "HRT", "Insulin sensitizers", "Statins".

Note S3. Examples of the data pre-processing for selected variables

On top of the 344 selected traits, we did a QC step as:

1. Added key variables
2. Processed ICD-10 coded health-related traits
3. Removed non-analysable variables
4. Handled variables with multiple arrays
5. Addressed low-frequency categories
6. Filtered out variables with high missing rate.

Specifically,

1. Added key variables

We added 8 traits, including education, BMI, hypotension, carotid atherosclerosis, TBI, anti-inflammatories, phenoAge and gender for the network analysis.
That resulted with 352 variables, among which 146 were health-related traits (either source or date of diagnosis of diseases, containing traits information of 80) 206 were baseline traits.

2. Processed ICD-10 coded health-related traits

(1) For ICD10 Coded health-related traits (n =80):
  a. Extract the diagnosis date of each disease.
  b. Compare the date with the date attending assessment centre for each disease.
  c. If the date is earlier than the date attending the assessment centre, mark the disease as 1, otherwise as zero.

(2) For other baseline traits:

3. Removed non-analyzable variables

  a. Removed date, time and image variables (n =7, variable UKB Code: 30761, 105010, 105030, 110006, 20222, 20223, 20226)
  b. Removed traits that are not present in baseline or present only in pilot study (n=8, variable UKB Code:10005, 10115,10818, 10912, 10962, 10971, 21765, 26302)

4. Handled variables with multiple arrays

  c. Variables that have multiple arrays:
     i. Variable UKB Code 84 (age or year cancer diagnosed) was dichotomized as 'diagnosed before recruitment or not'.
     ii. Variable UKB Code 22611, 22614 (occupational health), one participant have 40 arrays of different answers: A participant was considered to have been exposed frequently if they answered "Often" and/or "Sometimes" at least once over 40 arrays [4].
  d. Compound variable (UKB Code 40042, Sleep – Day average): a set of values required as a whole to describe some compound property. For each cell, the mean value of each value set was taken.
  e. The smoking status variables that are based on current or previous smoking status had dichotomised them by current or previous smoking status. (n= 10, UKB Code: 1239, 1249, 6157, 6158, 2867, 2897, 3436, 6194, 22507, 3496)
  f. Variables with multiple single values (multiple choices):
     i. Questions indicating certain situations: (n=5, UKB Code: 6152, 6154, 6164, 20001, 6138) Dichotomise them into binary variables.
     ii. Questions followed by binary-choice question (n=1, UKB Code: 20548): Extract the information from the former question.

iii. Questions that cannot be transferred: (n =1, 20086): Removed

g. Other situations:
Variables followed by binary-choice question (e.g., age of disease diagnosis): n=8, variable UKB Code: 2976, 3627, 4022, 22150, 22155, 4056, 22160, 2986): we used the information from the former questionnaire.
Remove: 2976, 3627, 4022, 22150, 22155, 4056, 22160, 2986
Using: 2443, 22130, 22135, 22140, 6152(multiple) add 6150 (added)

Among the former questions, UKB Code: 2443 for 2976,2986; 22130 for 22150, 22135 for 22155, 22140 for 22160, 6152 for 4022 were already exist in the list. Adding UKB Code:6150 for 3627, 4056

5. **Addressed low-frequency categories**

h. The variables with extreme small numbers (n <50) in categorical levels were checked. We found the extreme numbers in online-follow up questions are caused by 'not sure'. To avoid problems caused by this level, we dichotomised all online-following questions of diseases (n =8, Variable UKB Code 120008, 120079, 2345, 20446, 120001, 120002, 21068, 20406).

i. The outcome variable (dementia diagnosis) was classified into three categories:
   1. diagnosed within two years of recruitment.
   2. diagnosed after two years of recruitment.
   3. not diagnosed

(3) 256 variables remained in the tidied cohort.

6. **Filtered out variables with high missing rate.**

(4) Visualize missing pattern before imputation (Figure S7):

(5) Final Filter by missing pattern >0.75 (n=109), column with only one count value (n=1), count variables with number smaller than 50 (n=23)
   (a) In parallel analysis:
      (i) adding phenotypic age [5], [6] related variables (n=9).
      (ii) Sample QC: removed outlier sample with 'infinite' phenotypic age (n=406,781)
      (iii) Sample cohort (sample size of 406,781) with 132 variables, including dementia and ID.
      (iv) After imputation, 2 variables were consistently not imputed (V16, V28), and being removed. Ten variables used to construct phenotypic age were removed.
      (v) 121 variables are putted into network analysis, including dementia.

(6) Sample cohort (sample size of 406,781) with 123 variables, including dementia and ID. After imputation, 2 variables were consistently not imputed (V16, V28), and being removed.

(7) 121 variables are put into network analysis.

Note S4. Examples of sample quality control and the study cohort:

502,371 participants

- remove related individuals (UKB Code: 22020, n= 95,357)
- removed unmatched sex (UKB Code: 22001& 31, n= 372, fully overlapped with related individuals)
- diagnosed with dementia before recruitment (UKB Code 42018 & 42019, n =177)
- removed participants with extreme phenotypic age [6] values (n = 56)

406,781 participants were included in the analysis, among which 97 individuals diagnosed with dementia within two years of recruitment and 5,954 were diagnosed above two years. In the cohort, 219,457 (53.9%) were women; mean (SD) age at recruitment was 56.5 (8.1) years.

Note S5. Illustrations of the causal discovery approaches used in the study

FCI algorithm [7] is a constraint-based causal discovery technique designed to search for causal relationships in the presence of potential unobserved confounders. This capability is achieved through extensively testing for conditional independencies within the data. When FCI encounters a scenario where no observed structure patterns can explain the association between two variables, it suggests the possibility of latent factors influencing these associations. This feature makes FCI particularly powerful for inferring causal relationships from observational data, especially in situations where the causal sufficiency assumption — the belief that all confounders are included in the dataset — is difficult to satisfy. Nevertheless, the FCI algorithm's requirement for extensive independence tests can be computationally demanding, limiting its scalability for large datasets. Therefore, FCI in combination with rapid structural learning techniques, such as Mixed Graphical Models (MGM) [8] were recommended for more efficient causal inference in big data [9].

The skeleton structure inferred from MGM was used as prior knowledge to inform the initial structure in the FCI algorithm. Mixed Graphical Models (MGM) is a structural learning algorithm that handles mixed data types in a relatively short time. The skeleton structure inferred from MGM, which previously have been proven to be efficient in help with FCI without sacrificing on the performance, was used as prior knowledge to inform the initial structure in the FCI algorithm [9].

Note S6. Interpretation of edges output from FCI algorithm [10]

The output graph of FCI will have four types of edges around variables, the interpretations are:

A -> B: A is the cause of B

A o-> B: A is a cause of B, or there is an unobserved confounder between A and B

A <-> B: There is an unobserved confounder between A and B

A o-0 B: Any of the situations:

- A is a cause of B
- B is a cause of A
- there is an unobserved confounder between A and B
- both situation 1 and 3
- both situation 2 and 3

Note S7. Python code for the variable selections

```
# -*- coding: utf-8 -*-
"""
@author: Xinzhu
"""
###import packages
#################
import gensim
import gensim.downloader as api
import numpy as np
from nltk.tokenize import word_tokenize
from nltk.corpus import stopwords
import nltk
nltk.download('stopwords')
nltk.download('punkt')
import re
import pandas as pd
from scipy.spatial.distance import cosine
from gensim.models.doc2vec import Doc2Vec
stop_words = set(stopwords.words('english'))  ##ignore words that does not add meaning to english

####################################################
###read excel that can be downloaded at 'https://biobank.ctsu.ox.ac.uk/~bbdatan/Data_Dictionary_Showcase.csv'
######################################################
base_col = pd.read_excel("Dictionary_Showcase_filtered.xlsx")

##define our dementia aimed phrase
dementia_phrases = ["Dementia","Alzheimer's disease","Cognitive decline","Memory loss",
                "Education","Bilingualism","Alcohol","Cognitive engagement","Diet",
                "Physical activity","Sleep","Smoking","Social engagement","Stress","Arthritis",
                "Atrial fibrillation","Anxiety","BMI","Cancer","Carotid atherosclerosis","Cholesterol",
                "Depression","Diabetes","Hearing loss","Homocysteine","Hormones","Hyper/hypotension",
                "Inflammatory markers","Metabolic syndrome","Motor function","Peripheral artery disease",
                "Renal disease","Serum uric acid","Stroke","TBI","Antacids","Antihypertensives",
                "Anti-inflammatories","Benzodiazepines","HRT","Insulin sensitizers","Statins","Pesticides"]
dementia_phrases = [phrase.replace('/', ' or ') for phrase in dementia_phrases]
dementia_tokens = [word.lower() for phrase in dementia_phrases for word in word_tokenize(re.sub(r'[^\w\s]','',phrase)) if word.lower() not in stop_words and word.isalnum()]

phrases =list(base_col['Field'])

num_runs = 1000  # Number of runs to adjust the random uncertainty caused by the algorithm
# New: loop over models
# pre-trained models are downloaded from: https://github.com/jhlau/doc2vec#pre-trained-doc2vec-models
doc2vec_models = {'model1': Doc2Vec.load('doc2vec/enwiki_dbow/doc2vec.bin'),
               'model2': Doc2Vec.load("doc2vec/apnews_dbow/doc2vec.bin")}

results = []

# Loop over models
for model_name, model in doc2vec_models.items():
    snr_results = []
    dementia_vector = model.infer_vector(dementia_tokens)

    # Loop over all the phrases
    for i, phrase in enumerate(phrases):
        scores = []

        # Loop for the specified number of runs
```

```
        for run in range(num_runs):

            print(model_name,"Phrase Index", i,run+1)
            # Preprocess and tokenize the phrase
            phrase_tokens = [word.lower() for word in word_tokenize(re.sub(r'[^\w\s]','',phrase)) if word.lower() not
in stop_words and word.isalnum()]

            # Infer the doc2vec vector for the phrase
            phrase_vector = model.infer_vector(phrase_tokens)

            # Compute the cosine similarity between the phrase and dementia_phrase vectors
            similarity_score = np.dot(phrase_vector, dementia_vector) / (np.linalg.norm(phrase_vector) *
np.linalg.norm(dementia_vector))

            # Add the score to the list
            scores.append(similarity_score)


        # Calculate mean and standard deviation
        mean_score = np.mean(scores)
        std_score = np.std(scores)

        # Calculate the signal-to-noise ratio
        snr = mean_score / std_score if std_score > 0 else 0

        # Add the SNR to the results
        snr_results.append({'Model': model_name, 'Phrase': phrase, 'Scores': scores, 'Mean': mean_score, 'Std':
std_score, 'SNR': snr})

    # Combine all SNR results for each model
    results += snr_results

# Convert the SNR results to a DataFrame and save to a CSV file
results_df = pd.DataFrame(results)
results_df.to_csv('doc2vec_final_results.csv', index=False) ##save results to local file in csv format


####################################
##word2vec
model_names = ['word2vec-google-news-300', 'glove-wiki-gigaword-300', 'glove-twitter-200', 'fasttext-wiki-
news-subwords-300']
models = [api.load(model_name) for model_name in model_names]

similarity_scores_list = []

for model in models:
    similarity_scores = []

    for phrase in phrases:
        max_similarity_score = -1  # Initialize the max similarity score to -1

        for dementia_phrase in dementia_phrases:
            # Tokenize and preprocess the phrase and dementia_phrase
            phrase_tokens = [word.lower() for word in word_tokenize(re.sub(r'[^\w\s]','',phrase)) if word.lower() not
in stop_words and word.isalnum()]
            dementia_tokens = [word.lower() for word in word_tokenize(re.sub(r'[^\w\s]','',dementia_phrase)) if
word.lower() not in stop_words and word.isalnum()]

            # Calculate average embeddings for each phrase using only valid word embeddings
            valid_phrase_embeddings = [model[token] for token in phrase_tokens if token in model]
```

```
        valid_dementia_embeddings = [model[token] for token in dementia_tokens if token in model]

        if valid_phrase_embeddings and valid_dementia_embeddings:
            phrase_embedding = np.mean(valid_phrase_embeddings, axis=0)
            dementia_embedding = np.mean(valid_dementia_embeddings, axis=0)

            # Compute the cosine similarity between the phrase embedding and the dementia embedding
            similarity_score = np.dot(phrase_embedding, dementia_embedding) / (np.linalg.norm(phrase_embedding) * np.linalg.norm(dementia_embedding))
            max_similarity_score = max(max_similarity_score, similarity_score)  # Update the max similarity score

      similarity_scores.append(max_similarity_score)  # Append the max similarity score to similarity_scores

   similarity_scores_list.append(similarity_scores)

# Create a Pandas DataFrame with the similarity scores for each model as columns
results_df2 = pd.DataFrame({'Phrase': phrases})
for i, model_name in enumerate(model_names):
   results_df2[f'Max Similarity Score ({model_name})'] = similarity_scores_list[i]

# Calculate the overall average score over the four models for each phrase
results_df2['Overall Average Score'] = results_df2[[f'Max Similarity Score ({model_name})' for model_name in model_names]].mean(axis=1)

results_df2.to_csv('word2vec_final_results.csv', index=False) ##save results to local file in csv format
```

Note S8. R code for imputation and network analysis

```
#!/opt/apps/apps/gcc/R/4.1.0/bin/Rscript
library(mice)
library(parallel)
library(rCausalMGM)
library(tidyverse)
library(dplyr)

dir <- Sys.getenv("DIR") # Get the environment variable DIR representing the directory path ##in total we have 50 directories
print(c('dir:',dir))

# Load the cohort data before imputation
load('my_cohort.rdata')

################################################################################
# Extract the file number from the directory path for use in setting the seed
number <- as.numeric(sub("FULL DIRECTORY/imp_", "", dir))
print(number)

set.seed(number) # Setting seed for reproducibility
print(c('seed_number: ', number))

# Perform MICE imputation using random forest with 5 iterations ##repeated for 50 times
# The first column is ID, therefore it is removed
time_taken <- system.time({
  mice_output <- mice(my_cohort[,2:132], method = 'rf', m=1, maxit = 5)
})
print(c('rfimp_time:', time_taken))
save(mice_output, file = paste0(dir,'/rf_output.rdata'))

# Extract complete data from MICE output
imp_data <- complete(mice_output)  ###complete datasets for analysis

############################################
# Quality control (QC) for imputed dataset
# Check and remove columns with only one unique value
one_value_columns <- names(imp_data)[sapply(imp_data, function(x) length(unique(x)) == 1)]
imp_data <- imp_data %>% select(-one_value_columns)

# Check and remove columns with missing values after imputation
na_columns <- names(imp_data)[sapply(imp_data, function(x) any(is.na(x)))]
print(c(" na_columns:", na_columns))
imp_data <- imp_data %>% select(-na_columns)
# Confirm ID consistency with original my_cohort
print("ID_identical_mycohort&impcohort :")
print(identical(imp_data[,c("Y","V15")], my_cohort[,c("Y","V15")]))

# Save the processed imputed data
save(imp_data, file = paste0(dir,'/original_imp.rdata'))

###standardisation numeric columns
imp_num <- imp_data %>%
  select(where(is.numeric))
imp_num <- scale(imp_num)
imp_fac <- imp_data %>%
  select(where(is.factor))
print(identical(imp_data[,c("V30","Y")],imp_fac[,c("V30","Y")])) ###double ensure the columns are consistent
```

```
imp_norm <- cbind(imp_num,imp_fac)
imp_data <- imp_norm
save(imp_data, file = paste0(dir,'/imp_data.rdata')) ###data for network analysis

##########################################################
########## Conduct analysis ##################################
variables <- colnames(imp_data)

# Assuming 'Y' is the variable to be restricted
restricted_variable <- "Y"   ###dementia
# Assuming all variables are named V1, V2, ..., Vn
all_variables <- variables
# Generate forbidden edges
forbidden_edges1 <- lapply(all_variables[all_variables != restricted_variable], function(variable) {
  c(restricted_variable, variable)
})

restricted_variable2 <- "V30"  ### gender
forbidden_edges2 <- lapply(all_variables[all_variables != restricted_variable2], function(variable) {
  c(variable, restricted_variable2)
})

restricted_variable3 <- "V12"  ##age at recruitment
forbidden_edges3 <- lapply(all_variables[all_variables != restricted_variable3], function(variable) {
  c(variable, restricted_variable3)
})

knowledge <- list(
  forbidden = c(forbidden_edges1,forbidden_edges2,forbidden_edges3))


time_taken <- system.time({
  mgm_fit <- mgm(imp_data)

})
print(c('mgm:',time_taken))

pdf(paste0(dir,'/mgm.pdf'))
plot(mgm_fit)
dev.off()
save(mgm_fit,file = paste0(dir, "/mgm_fit.rdata"))

####FCI analysis###

time_taken <- system.time({
  fit_fcimax <- rCausalMGM::fciStable(imp_data,initialGraph = mgm_fit,alpha=0.05,
                                  fdr= TRUE, orientRule = 'maxp',knowledge =knowledge )

})

print(c('fci:',time_taken))
save(fit_fcimax, file = paste0(dir, "/fci_max.rdata"))
```

References

[1] T. Mikolov, K. Chen, G. Corrado, and J. Dean, ‘Efficient Estimation of Word Representations in Vector Space’, Sep. 06, 2013, *arXiv*: arXiv:1301.3781. doi: 10.48550/arXiv.1301.3781.

[2] J. H. Lau and T. Baldwin, ‘An Empirical Evaluation of doc2vec with Practical Insights into Document Embedding Generation’, Jul. 18, 2016, *arXiv*: arXiv:1607.05368. doi: 10.48550/arXiv.1607.05368.

[3] K. J. Anstey, N. Ee, R. Eramudugolla, C. Jagger, and R. Peters, ‘A Systematic Review of Meta-Analyses that Evaluate Risk Factors for Dementia to Evaluate the Quantity, Quality, and Global Representativeness of Evidence’, *Journal of Alzheimer's Disease*, vol. 70, no. s1, pp. S165–S186, Jan. 2019, doi: 10.3233/JAD-190181.

[4] H. A. Amin, P. Kaewsri, A. M. Yiorkas, H. Cooke, A. I. Blakemore, and F. Drenos, ‘Mendelian randomisation analyses of UK Biobank and published data suggest that increased adiposity lowers risk of breast and prostate cancer’, *Sci Rep*, vol. 12, p. 909, Jan. 2022, doi: 10.1038/s41598-021-04401-6.

[5] M. E. Levine *et al.*, ‘An epigenetic biomarker of aging for lifespan and healthspan’, *Aging (Albany NY)*, vol. 10, no. 4, pp. 573–591, Apr. 2018, doi: 10.18632/aging.101414.

[6] Z. Liu, P.-L. Kuo, S. Horvath, E. Crimmins, L. Ferrucci, and M. Levine, ‘A new aging measure captures morbidity and mortality risk across diverse subpopulations from NHANES IV: A cohort study’, *PLOS Medicine*, vol. 15, no. 12, p. e1002718, Dec. 2018, doi: 10.1371/journal.pmed.1002718.

[7] P. Spirtes, C. N. Glymour, and R. Scheines, *Causation, Prediction, and Search*. MIT Press, 2000.

[8] A. J. Sedgewick, I. Shi, R. M. Donovan, and P. V. Benos, ‘Learning mixed graphical models with separate sparsity parameters and stability-based model selection’, *BMC Bioinformatics*, vol. 17, no. 5, p. S175, Jun. 2016, doi: 10.1186/s12859-016-1039-0.

[9] V. K. Raghu *et al.*, ‘Comparison of strategies for scalable causal discovery of latent variable models from mixed data’, *Int J Data Sci Anal*, vol. 6, no. 1, pp. 33–45, Aug. 2018, doi: 10.1007/s41060-018-0104-3.

[10] X. Shen, S. Ma, P. Vemuri, and G. Simon, ‘Challenges and Opportunities with Causal Discovery Algorithms: Application to Alzheimer's Pathophysiology’, *Sci Rep*, vol. 10, no. 1, Art. no. 1, Feb. 2020, doi: 10.1038/s41598-020-59669-x.
